# Dynamic polarization vision in mantis shrimps

**DOI:** 10.1038/ncomms12140

**Published:** 2016-07-12

**Authors:** Ilse M. Daly, Martin J. How, Julian C. Partridge, Shelby E. Temple, N. Justin Marshall, Thomas W. Cronin, Nicholas W. Roberts

**Affiliations:** 1 School of Biological Sciences, University of Bristol, Tyndall Avenue, Bristol BS8 1TQ, UK; 2 School of Animal Biology and the Oceans Institute, University of Western Australia, 35 Stirling Highway (M317), Crawley, Western Australia 6009, Australia; 3 Queensland Brain Institute, The University of Queensland, St Lucia, Queensland 4072, Australia; 4 Department of Biological Sciences, University of Maryland Baltimore County, 1000 Hilltop Circle, Baltimore, Maryland 21250, USA

## Abstract

Gaze stabilization is an almost ubiquitous animal behaviour, one that is required to see the world clearly and without blur. Stomatopods, however, only fix their eyes on scenes or objects of interest occasionally. Almost uniquely among animals they explore their visual environment with a series pitch, yaw and torsional (roll) rotations of their eyes, where each eye may also move largely independently of the other. In this work, we demonstrate that the torsional rotations are used to actively enhance their ability to see the polarization of light. Both *Gonodactylus smithii* and *Odontodactylus scyllarus* rotate their eyes to align particular photoreceptors relative to the angle of polarization of a linearly polarized visual stimulus, thereby maximizing the polarization contrast between an object of interest and its background. This is the first documented example of any animal displaying dynamic polarization vision, in which the polarization information is actively maximized through rotational eye movements.

## Abstract (web summary)

Mantis shrimps are known to display large pitch, yaw and torsional eye rotations. Here, the authors show that these eye movements allow mantis shrimp to orientate particular photoreceptors in order to better discriminate the polarization of light.

## Graphical abstract

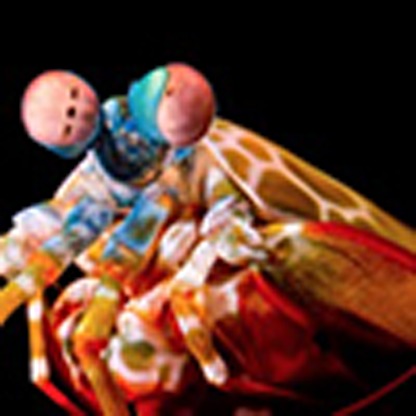

Many animals go to great lengths to stabilize their gaze, using eye, head and body movements to avoid motion blur^1^. Blowflies^2^, pigeons^3^, crabs^4^,^5^ and cats^6^ are a few diverse examples of animals from distinct orders known to do this. In these examples gaze stabilization enhances the ability to both detect objects, especially those occupying a small area of the visual field, and to estimate relative motion within a visual scene^7^. In addition, by stabilizing gaze, an animal can maintain its orientation relative to a local horizon^4^ or other salient landmark. The head, body or eye movements employed as gaze stabilization mechanisms in all of the above examples are reactive, products of the vestibular-ocular and optokinetic reflexes^1^.

Three types of rotation, pitch, yaw and torsional (roll), are all used as eye movements to stabilize an animal's gaze. Torsional rotations are most relevant for animals with lateral eyes, such as ungulates and lagomorphs, whose modes of locomotion involve considerable angular displacements of the head. In such species, large head rotations are compensated by correspondingly large counter-rotations of the eyes^8^,^9^. In humans, and other animals with frontal eyes, stabilizing eye movements are more evident in the pitch and yaw planes of rotation relative to the head's principal axes^1^,^10^,^11^. Nevertheless, small involuntary torsional (also referred to as cyclotorsional) eye movements can still occur in these animals depending on the rotations of the head or body^12^,^13^,^14^,^15^,^16^,^17^. Small (<5°) torsional rotations have also been reported after horizontal saccades^18^ and during vergence^19^,^20^ in human subjects. These torsional rotations are thought to be secondary consequences of the action of ocular muscles.

While all these motions are reactive behaviours, there is one animal group that exhibits very unusual and particularly large pitch, yaw and torsional eye rotations as a proactive strategy during visual search. Stomatopods (commonly known as mantis shrimps) are an unusually inquisitive group of stalk-eyed crustaceans with an extensive repertoire of eye movements. With these large ranges of pitch, yaw and torsional rotations, their eyes can exhibit coordinated or independent tracking^21^, object of interest acquisition through saccades^22^ and scanning motions^1^,^23^. Of these eye motions, particularly noteworthy are the proactive torsional rotations of up to 90° (refs 23, 24); (Fig. 1a–d), which again can be either coordinated or completely independent as required^21^,^23^,^24^ (Fig. 1c,d).

In addition to their intricate eye movements, stomatopods also have an extraordinarily complex visual system and the structure of their eyes is especially unusual. Each of their apposition compound eyes is divided into three sections: two hemispheres bisected by a narrow linear midband two or six ommatidial rows wide (depending on the species)^25^,^26^,^27^,^28^,^29^,^30^ (Fig. 1b). Particular to each of these three sections are intra-retinal adaptations to the basic photoreceptor anatomy, which have enabled stomatopods to evolve regional specializations for both linear and circular polarization vision^25^,^31^,^32^, as well as 12-channel colour vision^32^,^33^,^34^. In the six row midband of *Odontodactylus scyllarus* *and Gonodactylus smithii*, the photoreceptors of the top four rows are regionally specialized for colour vision, while rows five and six are specialized for polarization vision^25^,^31^,^32^. In some species, rows five and six also show adaptations for circular polarization sensitivity. The proximally placed ultraviolet-sensitive R8 cells can act as quarter wave plates^35^, converting circularly polarized light into linearly polarized light, which can then be analysed by the underlying R1-7 cells. Stomatopods have also evolved an array of visual signals that contain both polarization and colour information. In the context of their eye movements, Land *et al*.^23^ demonstrated that *O. scyllarus* also possess a scanning-like motion in which the eyes perform relatively slow pitch rotations (40° s^−1^) as scans in a plane approximately perpendicular to the mid-band, theoretically providing two-dimensional colour information by scanning a one-dimensional row of assigned colour channels. This sequential analysis is equivalent to man-made ‘push broom' spectral sensors. Importantly, the slow speed of these scans differentiates these movements from typical visual saccades.

While eye-scanning movements suggest a functional link between the stomatopod's eye anatomy and colour vision, the reasons behind the extensive torsional rotations of the eyes remain unknown. One possibility is that these rotations are linked to the animal's polarization vision. Typically, in most polarization vision systems, the animal is unable to vary the orientation of their polarization receptors relative to a signal in a controlled manner. Torsional eye movements could, in principle, allow stomatopods to rotate the position of a group of polarization-sensitive microvilli to maximize the information available from a polarized scene^25^,^36^,^37^. In analogy, this is comparable to rotating a piece of Polaroid filter in front of a camera to find the best angle to reduce polarized glare.

We therefore hypothesize that any torsional rotations that occur when a stomatopod visually inspects a polarized stimulus would act to align the closest group of microvilli in the hemispheres relative to the polarization angle of the stimulus, thereby maximizing the perception of the polarization signal. This effectively acts to maximize the polarization contrast. Alignment of the closest group of microvilli requires the least ocular torsion and is therefore likely to be dynamically and energetically most efficient. In the set of experiments reported here we test this prediction and ask three questions: (1) Do stomatopods rotate their eyes in response to a linearly polarized stimulus? (2) Do they align specific photoreceptors relative to the polarization of that stimulus? and (3) Do the rotations maximize the polarization distance (PD) of the signal versus the background? To answer these questions, we track in three dimensions the eye movements of two species of stomatopod, *G. smithii* and *O. scyllarus*.

## Results

### Microvilli angles

**Figure 2:**
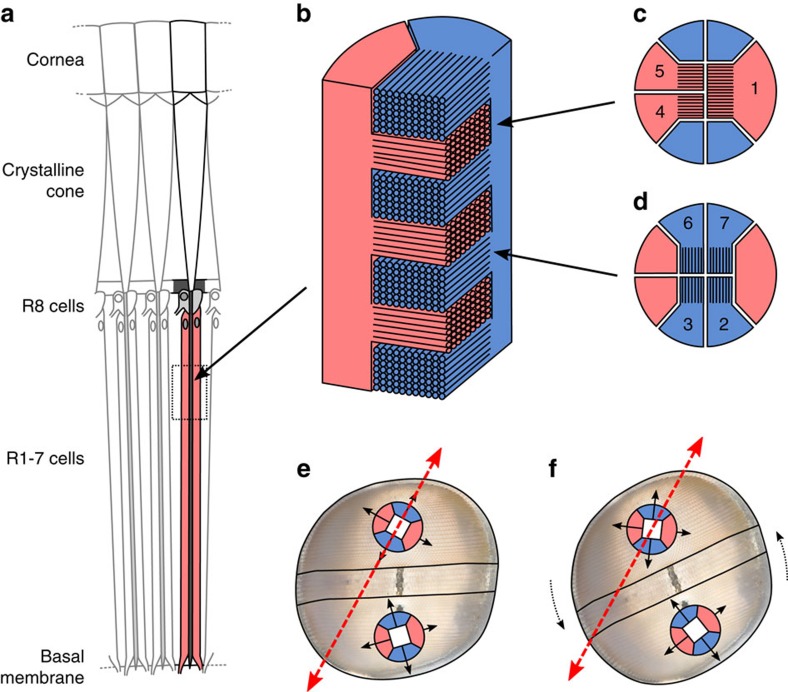
Diagrams illustrating the polarization anatomy of a mantis shrimp eye and the relevant geometries. (**a**) Longitudinal section through a stomatopod eye hemisphere showing the main rhabdom (retinular (R) cells 1–7) and the distal R8 cell (redrawn from Marshall *et al*.^26^). (**b**) Bi-directional microvillar projections from two retinular cells (coloured red and blue for illustrative purposes) forming stacked layers within the main rhabdom (similar to standard crustacean eye anatomy). (**c**,**d**) the orientation of microvilli in alternate layers of the main rhabdom. (**e**) Relative orientations of microvilli in the main rhabdoms in each hemisphere (note that these are not drawn to scale). There is a 45° skew in the overall orientation of the microvillar directions between the dorsal and ventral hemispheres. In **e**, the dorsal microvilli are optimally aligned for detecting the incoming polarized light stimulus (dashed red arrow; that is, effective angle *ϕ*=0°). In **f**, the eye has rotated by 22.5° with the consequence that both sets of orthogonal microvilli are maximally misaligned with the stimulus (that is, *ϕ*=22.5°). (**b**–**d**) Redrawn from Goldsmith^57^. Background image in **e** and **f** adapted from a photo by R Caldwell.

To understand how torsional eye rotations affect polarization vision in a stomatopod, it is important to learn how changes in eye position alter the orientation of sets of microvilli within the photoreceptors. The dorsal and ventral hemispheres of the stomatopod eye have typical crustacean photoreceptors with layered, interdigitating, orthogonal projections of microvilli (Fig. 2a–d). Because the highest receptor sensitivity occurs when the angle of polarization is oriented parallel to the long axis of the microvilli^25^,^38^,^39^, the rhabdom of each ommatidium has two polarization-sensitive channels, with peak sensitivity to perpendicular orientations of the angle of polarization^25^,^27^,^28^,^29^,^30^. However, across the whole eye the orientations of these two channels differ between the dorsal and ventral hemispheres, with the microvillar orientations of each half of the eye offset by 45° (Fig. 2e). As a result, in the two hemispheres there are four sets of microvilli in total with their maximum sensitivities separated by 45°, and as a consequence, an eye is never more than 22.5° away from aligning one group of microvilli with a specific angle of polarization. The periodic nature of this functional alignment suggests that any eye only has a single effective angle (*ϕ*) through which it must rotate to line up any of the four groups of microvilli with the stimulus. This effective range is 0°≤*ϕ*≤22.5°, where *ϕ*=0° corresponds to perfect alignment (Fig. 2e). At perfect misalignment, all four orientations of the microvilli in either hemisphere are oriented at an effective angle of *ϕ*=22.5° away from the angle of polarization of the stimulus (Fig. 2f).

### Measuring polarization distance

To quantify the level of the polarization signal that may be detected by the animal, we used the framework developed by How and Marshall^40^ to calculate values of PD for both the dorsal and ventral receptor pairs (see Methods for a full derivation). In analogy with the concept of colour distance in a colour space, PD provides a quantitative measure of the perceived contrast between two polarization states for a model visual system. A greater polarization distance indicates a greater level of perceived polarization contrast between two stimuli. Unlike colour distance, PD may be maximized by torsional rotation of the receptors and their physical alignment with the angle of polarization of a stimulus.

In this context it is important to consider the polarization environment of stomatopods in their natural habitat and how rotating the eye could maximize the PD of, for example, polarized signals against the unpolarized body parts of conspecifics. Underwater scenes vary in the background level of polarization. Typically, the benthic substrate reflects very little polarization (<5%), while the underwater space light tends to be linearly polarized between 10 and 50% depending on both the sun's overhead position and the water clarity^41^. Therefore, the polarization of inter- and intra-specific visual signals are commonly viewed against either unpolarized or partially polarized backgrounds (that is, if the signal is viewed against the benthos or against open water, respectively)^31^,^42^,^43^,^44^,^45^. A key consideration here is that the torsional position of the eye required to maximize the PD depends on both the polarization of the background and of the stimulus. When the background is unpolarized, the maximum PD occurs when a group of microvilli are aligned parallel to the polarization angle of the stimulus. However, if the background is polarized at an angle different to the stimulus, then the position of maximum PD occurs when elements of a pair of receptors are each aligned as closely as possible to the angle of both the background and stimulus polarization. This leads to the prediction that, if an animal is using the torsional rotations to maximize the perceived PD then it will not simply align the microvilli with the polarization angle of the stimulus, but with the angle of the predicted maximum PD.

### Eyes rotate torsionally in response to linearly polarized stimuli

**Figure 3:**
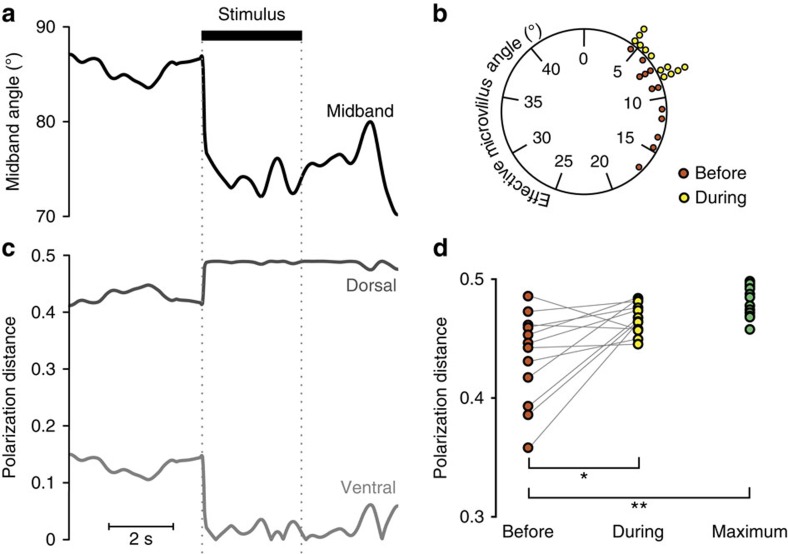
Experimental results for the LED experiment. (**a**) An example of the torsional rotation of the midband in response to the polarized LED stimulus (onset and offset of stimulus is denoted by black bar and dotted grey lines). (**b**) Angle subtended between the stimulus angle of polarization and the nearest polarization receptor before the onset (red circles) and during (yellow circles) the stimulus presentation. (**c**) An example of the polarization distance (PD) calculated for the dorsal (grey line) and ventral (light grey line) pairs of polarization receptors during a single stimulus presentation. (**d**) Same data as **b**, with paired comparison between the calculated PD, both before (red circles), and during (yellow circles) the stimulus presentation and compared with a set of bootstrap resampled maximum data points (green circles). Stars represent levels of statistical significance: *P<0.05; ^**^P<0.01; ^***^P<0.001.

*G. smithii* rotated their eyes in response to a single polarized light stimulus (Fig. 3a,b). Six wild-caught *G. smithii* were each shown eight presentations of a short duration (3 s) green LED stimulus (wavelength of maximum emission=501 nm) with a high percentage of linear polarization (*d*=99.5%), seen through a 4 mm aperture in an opaque black screen and the movements of their eyes were tracked using a stereo camera system. The angle of polarization was either vertical (*χ*=0°), or diagonal (*χ*=45°), with four trials of each. Since the two eyes of the stomatopod display a high degree of independence^11^,^23^, only data from the eye that reacted to the stimulus first was used in the analysis. The rotational responses were averaged across all four trials for each of the six animals. An initial torsional rotation of 15° (mean, 95% confidence intervals 10–20°, *n*=6) was observed, with a rotational speed of 45±8° s^−1^ (mean±s.d., *n*=6). The response rate was 83.33%. Secondary rotations of the eyes after the initial response to the stimulus were also measured and these were typically lower in amplitude (≈5°).

### Torsional rotations orient hemisphere microvilli

To analyse whether the perceived polarization contrast increased during these rotations, we calculated the PD between receptor pairs in both the dorsal and ventral hemispheres before and during the LED stimulus for both stimulus angles (*χ*=0°and *χ*=45°). Based on the mean variance in these experimental data, we also calculated a model data set (using a bootstrap resampling method) that represents an optimal alignment to maximize the PD. Before stimulus onset the PD was significantly different from the resampled model maximum PD values (Fig. 3c,d; repeated measures analysis of variance (RMANOVA), *F*=16.9, d.f.=1, *P*=0.001). However, during presentation of the stimulus the PD increased such that there was no longer a difference from the model maximum PD distribution (Fig. 3d: RMANOVA, *F*=5.4, d.f.=1, *P*=0.132). When viewing the stimulus, *G. smithii* significantly increased the PD by aligning one group of microvilli with the predominant angle of polarization of the linearly polarized stimulus (RMANOVA, *F*=9.5, d.f.=1, *P*=0.012). The angle of the polarization of the stimulus (*χ*=0° or *χ*=45°) was not a significant factor in any of the above measures (RMANOVA, *F*<2.7, d.f.=1, *P*>0.160) and there was no preference for maximizing the PD in either the dorsal or ventral hemispheres (binomial test, *P*=0.636). Importantly, the torsional rotations did not act to maximize the PD in the photoreceptors of rows five and six of the midband (RMANOVA, *F*=0.053, d.f.=1, *P*=0.821) and the response was not dependent on the angle of the polarization (RMANOVA, *F*=0.748, d.f.=1, *P*=0.400). Given the 501-nm wavelength of the stimulus, the role of the ultraviolet-sensitive R8 cells was not considered.

### Torsional rotations maximize the polarization contrast

The first experiment demonstrated that the torsional rotations of the eyes align the closest group of microvilli to the angle of a bright, polarized stimulus viewed against a dark unpolarized background, which consequently maximizes the PD. However, if the background is also bright and linearly polarized, aligning the effective angle with the polarization of the stimulus does not necessarily provide the greatest polarization contrast within the image. In such a situation, the maximum PD between the object of interest and the background would be achieved by orienting a pair of orthogonal microvilli so that one set is as close as possible to the background angle, and the other as close as possible to the stimulus angle. This optimum angle is also modulated in a predictable way by contrasts in degree of polarization between the stimulus and the background (see Methods for mathematical explanation of this theory). We investigated the response of stomatopods to polarized stimuli viewed against a differently polarized background to determine whether they use torsional rotations to maximize the polarization contrast.

Six *O. scyllarus* were presented with expanding circular looming stimuli displayed on an LCD monitor, which had been modified following the methods of How *et al*.^46^ to display polarization contrasts with no associated intensity contrasts. In addition, the inbuilt fluorescent light source of the monitor was removed, along with the plastic back of the monitor, and replaced with a back projecting bank of LEDs with a peak emission of 501 nm. As a result, different pixel values (on the RGB scale of 0–255) had different polarization characteristics, but a uniform colour and intensity. Animals were presented with a looming disc, which rapidly expanded (2.2 m s^−1^) from a single pixel to occupy a visual angle of 10°. The byte value of the pixels of the foreground circle were set to generate a difference in the angle of polarization of 61.2° between the circle and the background.

**Figure 4:**
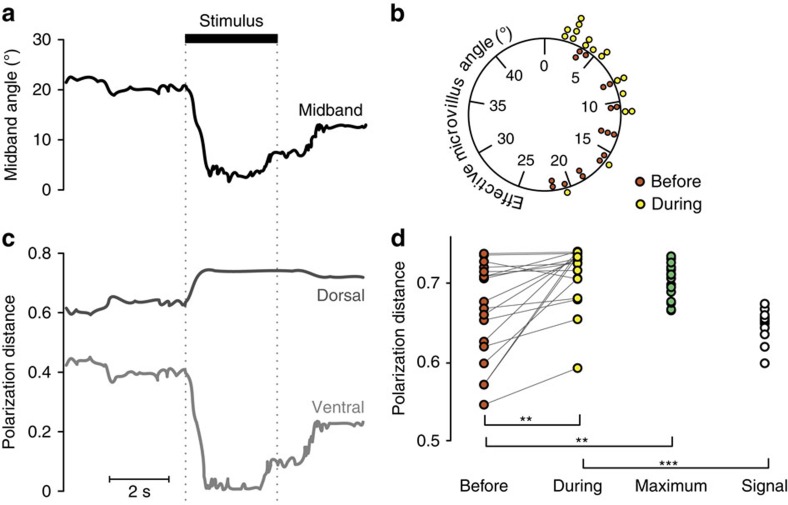
Experimental results where six *O. scyllarus* were presented with expanding circular looming stimuli displayed on an LCD monitor. Captions as for the LED experiment. Note that the angle measured in **b** is between the optimal receptor alignment for detecting the maximum polarization contrast, and the nearest set of polarization receptors. (**d**) Same data as **b**, with paired comparison between the calculated PD, both before (red circles), and during (yellow circles) the stimulus presentation and compared with a set of bootstrap resampled maximum data points (green circles). In addition, the modelled data set representing the viewed PD if the closest group of microvilli were aligned with the stimulus angle of polarization (open circles) is shown. Stars represent levels of statistical significance: *P<0.05; ^**^P<0.01; ^***^P<0.001.

From the angular rotations of the eyes, we again calculated the effective angles of the microvilli in each hemisphere and the associated PD (Fig. 4a–c). Based on the variance in the experimental data, we calculated a resampled bootstrap population of optimum rotation angles to maximize PD, against which to compare our data. As in the first LED experiment, the eye position was significantly different from the resampled maximal PD values before the onset of the stimulus (Fig. 4d; RMANOVA, *F*=8.6, d.f.=1, *P*=0.010). In response to the stimulus, the eyes rotated such that the PD no longer significantly differed from the resampled maximal PD distribution (Fig. 4d; RMANOVA, *F*=0.3, d.f.=1, *P*=0.688). During these rotations, *O. scyllarus* torsionally rotated their eyes to significantly increase PD after stimulus onset (Fig. 4d: RMANOVA, *F*=7.6, d.f.=1, *P*=0.009). These experiments were performed with the screen at three different tilt angles (0°, 6° and 14°), and in none of the above measures was tilt angle a significant factor (RMANOVA, *F*<1.4, d.f.=1, *P*>0.254). In 15 out of 18 trials, the PD was maximized in the ventral hemisphere, and in the dorsal hemisphere in the other three trials; a significant difference associated with an apparent five-fold dominance in the ventral hemisphere (binomial test, *P*=0.008). Moreover, the torsional rotations did not act to increase the PD in the photoreceptors of rows five and six of the midband (RMANOVA, *F*=2.281, d.f.=1, *P*=0.143) and the response was not dependent on the screen angle (RMANOVA, *F*=0.760, d.f.=1, *P*=0.391). It should be noted that the response rate of the animals during this experiment was 100%.

To further test whether the rotations aligned the eyes with either the maximum PD value or the polarization angle of the stimulus, we calculated a second set of data using the bootstrap method for the angle of polarization of the stimulus (Fig. 4d, white dots). Before the onset of the stimulus, the eye position was not significantly different from the PD values representing an alignment with the stimulus angle (Fig. 3h; RMANOVA, *F*=1.74, d.f.=1, *P*=0.234). During the stimulus presentation however, the rotations of the eye created an alignment that was significantly different (RMANOVA, *F*=40.3, d.f.=1, *P*<0.001). Therefore, we can conclude the rotations occur to maximize the PD of the signal against the background and not to simply align the closest microvillar group to the angle of polarization of the stimulus.

## Discussion

We have shown that two species of stomatopod, *G. smithii* and *O. scyllarus* exhibit torsional rotations of their eyes in response to linearly polarized stimuli; the first species to a single source of linear polarization, and the second to a pattern of polarization. In each experiment, the animals chose to maximize the polarization signal in one receptor pair. In the first experiment, PD_max_ was achieved by aligning the closest microvillar group with the angle of polarization of the stimulus, viewed against a dark unpolarized background. In the second experiment, the eye did not align with the angle of polarization of either the foreground or background, but to the torsional angle that maximized the contrast between the two. While the two experiments were carried out with different species, both *G. smithii* and *O. scyllarus* demonstrate the fundamental principle of enhancing polarization vision using dynamic torsional rotations. It is unlikely, given similarities between the eye morphology and the agonistic lifestyle of the two species that a behavioural hierarchy exists.

The concept of dynamic polarization vision has been discussed previously in the literature in the context of navigation using sky polarization patterns^37^,^47^. For example, the dung beetle *Scarabaeus rugosus* has been observed performing rotational dances on top of its dung ball before it attempts to roll away from the central heap and such whole-body movements could be involved in modulating the polarization signals received by the eye's dorsal rim area^48^. Similarly, it has been suggested that while navigating using the sky polarization pattern, desert ants perform whole-body rotational movements to scan the celestial hemisphere, serving a similar function^49^. However, to our knowledge, this is the first demonstration of dynamic polarization vision mediated by eye movements, and it is particularly interesting given its potential involvement in contrast enhancement for object detection and discrimination tasks.

One of the interesting findings of this work is the discovery that it is the hemispheres that play a key role in driving rotational eye movements, rather than the midband. In these two reported experiments, we found that the rotations did not increase the polarization contrast of the polarization sensitive photoreceptors in rows five and six of the midband as one perhaps would have expected based on the weight of the literature concerning midband polarization sensitivity in stomatopods. A puzzling aspect of the stomatopod eye is the 45° offset in microvillar alignment between the dorsal and ventral hemispheres. In all species in the Squilloidea, Lysiosquilloidea and Gonodactyloidea superfamilies examined, the orientation of the orthogonal pairs of microvilli from retinular cells R1-7 is rotated by 45° between the dorsal and ventral hemispheres, such that the microvilli are projected in four discreet directions across the eye in regions outside of the midband^25^. This differs from the compound eyes of every other crustacean studied to date, which have microvilli projected in two orthogonal directions only^38^,^50^,^51^. As with many features of the stomatopod eye, it seems unnecessary to have this added level of complexity, given that the eye can rotate over a range of 90° to adjust the orientation of the underlying receptors. One possible explanation for this architecture is that the hemispheres have large areas of overlap in their visual fields, particularly in the area of highest acuity. When investigating objects of interest, both sets of hemisphere receptors will receive visual inputs at the same time. If one hemisphere is oriented maximizing the polarization contrast, then the other hemisphere will be maximally misaligned with the stimulus (by default because of the 45° offset). At maximal misalignment, the hemisphere's receptors effectively receive no polarization information. As a result, the two hemispheres could then supply two independent channels of information, the former about polarization and the latter about intensity. In this way, stomatopods may have found an elegant method of avoiding confounds between the two visual dimensions of information.

A second and perhaps more plausible explanation is that this anatomy enables the animal to find the optimal polarization signal more quickly and unambiguously than a simple two-channel system. The 45° offset in receptor organization could speed the acquisition of the optimum polarization signal by reducing the effective angle range from 0°<*ϕ*<45°, to 0°<*ϕ*<22.5°, while minimizing the energetic cost of eye movements. This arrangement also removes the problem of null points of discrimination^37^,^40^,^51^, as at least one pair of receptors will always detect some relevant contrast from polarization cues. Given the fast-pace and hazardous nature of inter- and intra-specific interactions, this type of polarization vision could be particularly beneficial in the context of communication between individual mantis shrimps, particularly given that many species employ strongly linearly polarized body patterns for signalling^41^,^42^,^44^.

However, as an eye is never more than 22.5° away from an optimal alignment to maximize the viewed polarization contrast in the hemispheres, the extent of larger 90° range of rotations remains puzzling and suggests there may be another role for this large rotational range. The directions of the scanning movements involved in colour and circular polarization vision are in some way guided by visual features of the scene, and so torsional eye rotations could also serve to orient the eye for subsequent scans that involve yaw and pitch motions. Nevertheless, it must be noted that polarization may also be included as a visual feature of the scene, so may bias the scan orientation in some way.

In summary, the dynamic polarization vision system of mantis shrimps is yet another example of an exquisite and unique adaptation to visual perception in these crustaceans and such findings could prove useful for developing bio-inspired technology in the field of polarization cameras and image processing. However, there are still a number of questions about this remarkable polarization vision system that remain to be investigated, such as how do these animals determine which set of receptors (dorsal or ventral) to align with the stimulus?; how is the information processed downstream within the optic neuropil and beyond?; and how, if at all, is information combined from the two eyes?

## Methods

Adult *G. smithii* (carapace lengths varying between 40 and 70 mm) were collected by hand net during daylight hours from Coconut Reef, Lizard Island, Australia under Marine Parks permit G12/35042.1. Animals were held separately in small aquaria containing artificial burrows made from 20 mm diameter opaque plastic piping. Aquaria were illuminated with fluorescent lamps under a 12:12 h light:dark cycle and water was provided via the flow-through sea-water system at the Lizard Island Research Station (LIRS). Adult *O. scyllarus* (carapace lengths varying between 90 and 150 mm) were purchased from the marine trade (Tropical Marine Centre, Bristol, UK) and held in a self-contained salt-water aquarium facility at the University of Bristol under similar conditions. All experiments were conducted in accordance with University of Bristol, LIRS and University of Queensland codes of ethics for animal experimentation.

**Figure 5:**
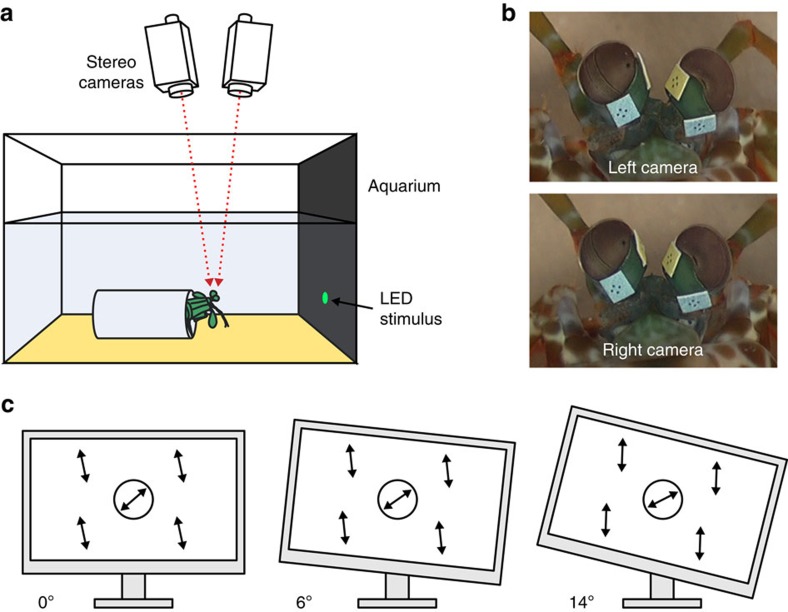
Experimental method. (**a**) Side view of the experimental apparatus for the LED experiment. (**b**) Frame-grabs from each video camera at a single point in time showing the pair of tracking markers fixed to each eye-stalk. (**c**) Illustration of the tilt positions of the modified LCD monitor for the second experimental setup and the consequent effect on loom and background angle of polarization.

Animals were lightly restrained using monofilament nylon fishing line wound around their thorax (avoiding restriction of the gills) within an artificial burrow (opaque flexible hose piping, diameter ca. 20 mm) with their heads and eyes protruding (Fig. 5a,b). They were placed in an experimental aquarium facing the stimulus and allowed to settle for 10–15 min.

### Visual stimuli

Animals were presented with two types of stimulus in separate experiments.

*LED experiment*. A green LED with a wavelength of peak emission at 501 nm (HLMP-CE34-Y1CDD, Avago Technologies, California, USA) was mounted behind a matt black card surface with a 4-mm diameter aperture (Fig. 5a). The LED wavelength was chosen to be near to the peak sensitivity of the photoreceptors in the stomatopod hemispheres^26^. A 20-mm square of linearly polarizing filter (American Polarizers, Reading, USA) combined with a diffuser (Teflon, DuPont, Delaware, USA) was mounted between the LED and the card aperture, so that the light transmitted through the aperture was close to 100% linearly polarized over the emission range of the LED. A microcontroller (Arduino Uno, Ivrea, Italy) connected to the MATLAB Arduino IO package (Mathworks, Massachusetts, USA) was used to control the LED. Each of six individual *G. smithii* was subjected to eight trials, consisting of a 5-s pre-stimulus period, followed by 3 s of LED on, and then 5 s of post stimulus. In four of the trials the angle of polarization was oriented vertically (*χ*=0°), and in the other four trials the orientation was diagonal (*χ*=45°). These two balanced treatments were shown in random order for each individual tested, with a randomly selected interval of between 45 and 90 s.

*Monitor experiment*. A Viglen (VL-15EC6, Viglen, UK) LCD monitor was modified by removing the front polarizer using the method of How *et al*.^46^, to produce polarization contrasts on the screen with no associated intensity contrasts (when viewed from a position orthogonal to the screen's surface). The inbuilt broadband fluorescent light source was removed and replaced with a back-projecting bank of 501 nm peak emission LEDs (as used in the LED experiment). A looming stimulus (generated using Psychtoolbox^52^,^53^) in which a disc rapidly (2.2 m s^−1^) expanded from a single pixel to a diameter of 35 mm (visual angle of 10°) was displayed to six *O. scyllarus.* The byte values of the pixels of the looming circle and the background were chosen such that they were matched in intensity differing by <0.2% across the visual range. The pixels of the background and circle matched both in ellipticity, *ɛ*, and the degree of polarization, *d*. The angle of polarization, *χ*, was set as shown in Table 1. Each animal was presented with six trials: three controls (in which looming stimulus pixels were of an equal value to the background) and three looming circle stimuli with pixel values as shown in Table 1. To test whether *O. scyllarus* use torsional rotations to improve contrast, rather than rotate to some preferred midband position, each of the three stimuli were presented with the screen tilted at three different angles: 0°, 6° and 14° (Fig. 5c).

### Eye tracking

During each trial, a stereo-camera system consisted of two video camcorders (Panasonic HC-X900, Osaka, Japan) recording the three-dimensional movement of both eyes. The two video camcorders, recording with a frame rate of 50 frames per second were mounted ca. 0.5 m above the experimental tank using a stereo camera mount and tripod. The focus of both cameras was set manually to maintain a fixed focal distance. Cameras were controlled using a single remote control and video recordings were synchronized to single video frame accuracy by the illumination of a 630-nm pilot LED positioned so as not to be visible to the animal. Small (2 × 2 mm square) tracking markers of white waterproof paper (Tyvek, DuPont, Delaware, USA) were fixed to the animals' eye stalks with cyanoacrylate glue (Fig. 5b). In all cases, animals were allowed to acclimatize to the markers for several hours before trials. Patterns on the markers consisted of four small points arranged in a cross, the position-of-which could be tracked automatically, frame-by-frame, for the duration of each trial.

A series of calibration images featuring checkerboards (8 × 10 squares) were used to calibrate the two camcorders to form a stereo pair (method modified from Bradski *et al*.^54^). Consequently, the location of the cameras with respect to the experimental apparatus could be determined and the distorting lens effects removed. The three-dimensional coordinates of a point visible in both of the cameras forming the stereo pair was determined using the pixel coordinates of that point in each of the camera's images and the transformation matrix computed during the calibration stage. Refraction effects caused by the water surface were incorporated using a mathematical correction based on the water depth and Snell's law. The relative positions of the markers were then used to calculate the three-dimensional position of the eyes. Torsional rotations of the eye were determined with an approximate measurement error of ±3°. This error was calculated during development of the method by comparing the calculated torsional poses with the actual poses of an artificial eye to which a set of tracking markers were attached. For analysis purposes, only one eye per animal was included in either data set and, if both eyes responded to the stimulus, the data relating to the eye that moved first were used.

### Polarization distance

The sensitivity of a particular group of microvilli to linearly polarized light depends on the effective angle, *ϕ*, which is given by


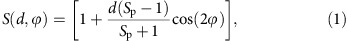


where *d* is the degree of linear polarization of the light and *S*_p_=10 is the polarization sensitivity of the photoreceptor^37^. The sensitivity of two perpendicularly orientated groups of retinular cells in each photoreceptor is then processed in an opponent manner, where the combined signal, *O*, from cell groups within a hemisphere can be approximated as the difference in the natural logarithm of the activity of the two receptor groups, *S*_I_ and *S*_II_ (ref. 37),


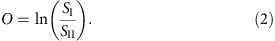


Polarization distance, PD, is an estimate of the polarization contrast between an object and its background as viewed by a model polarization vision system^40^. Roughly analogous to colour distance, PD calculates the level of contrast between two polarized stimuli based on a given photoreceptor arrangement. Mathematically, PD is proportional to the absolute difference between the opponency signals generated by the object, *O*_o_ and the background, *O*_b_,


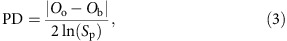


where *S*_p_ is the polarization sensitivity of the photoreceptor^37^. The maximum PD does not necessarily correspond to alignment with either the foreground or background polarization, but a compromise determined by the degree and the angular difference between the two, as given in equation (3).

### Statistical analysis

All statistical analyses were conducted in R 3.0.2 (ref. 55). Using the bootstrap method^56^ (*n*=10,000), normal distributions of virtual data (*rtnorm, base package)* with the same variance as the experimental values from ‘before' and ‘during' were calculated to represent the maximum PD values, and additionally for the LCD experiment, the PD when the closest group of microvilli would be aligned with the angle of polarization of the stimulus. Analyses used a repeated measure ANOVA test.

### Data availability

The data that support the findings of this study are available from the corresponding author upon request.

## Additional information

**How to cite this article:** Daly, I. M. *et al*. Dynamic polarization vision in mantis shrimps. *Nat. Commun.* 7:12140 doi: 10.1038/ncomms12140 (2016).

## Figures and Tables

**Figure 1:**
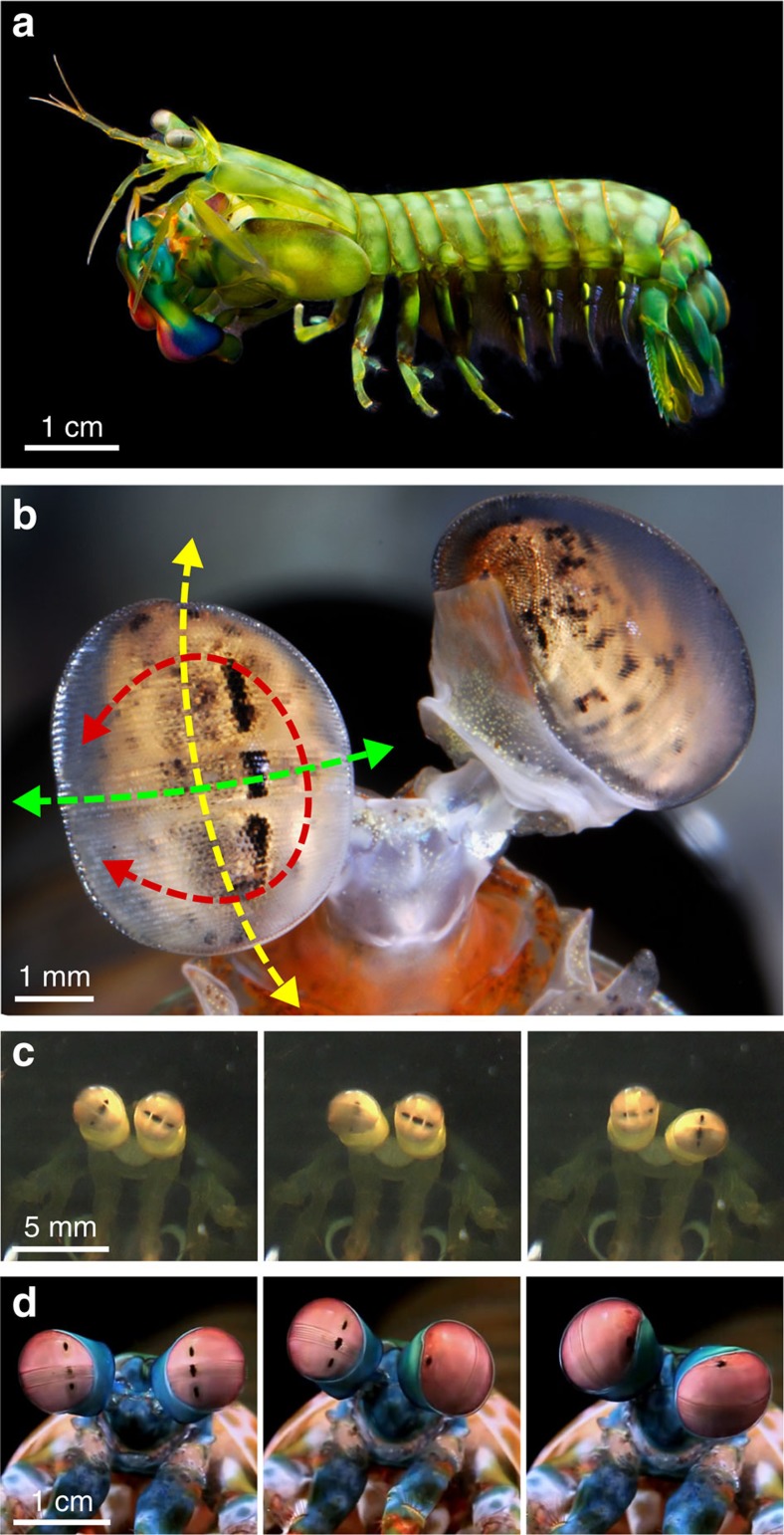
Mantis shrimp eye movements. (**a**) Side view of a *Gonodactylus smithii*. (**b**) Rotational degrees of freedom of stomatopod eyes relative to the external environment, as demonstrated in *Odontodactylus cultrifer*. Yellow arrows=pitch (up–down); green arrows=yaw (side-to-side); red arrows=torsional (roll) rotations. The midband is visible as a distinct stripe of ommatidial facets dividing the eye into dorsal and ventral hemispheres. (**c**,**d**) Series of video still frames demonstrating the torsional rotation range in *G. smithii* (**c**) and *Odontodactylus scyllarus* (**d**). (**c**) left eye - 45°, 85°, 0°; right eye - 30°, 20°, 90°; and (**d**) left eye - 90°, 80°, 0°; right eye - 90°, 0°, 90°.

**Table 1:** The pixel values at 501 nm illustrating the: predominant angle of polarization (* χ *), degree of polarization (*d*) and ellipticity (* ɛ *).

Value	Pixels	*χ* (°)	*d*	*ɛ*
Looming circle	[210 210 210]	49.06	0.99	0.23
Background	[0 0 0]	−12.19	0.92	0.23
